# Account of the Recent Epidemic Erysipelas in Delaware County, Pennsylvania

**Published:** 1844-09-07

**Authors:** Jesse Young

**Affiliations:** Chester, Pennsylvania


### ACCOUNT OF THE RECENT EPIDEMIC ERYSIPELAS IN DELAWARE COUNTY, PENNSYLVANIA.

By Jesse Young, M.D., of Chester, Pennsylvania.

During the past spring and early part of sum mer, our neighbourhood was visited by a severe form of erysipelas, of an epidemic character. I think for more than three months scarcely a case of disease occurred in my own practice, or in that of several of my neighbouring physicians, that did not, in some part of its course, manifest decidedly erysipelatous inflammation. It generally came on with the usual symptoms of catarrh; which frequently continuing some days before medical advice was sought, the patient would be attacked with a severe and protracted rigor, succeeded by fever and violent pains of the head, back and bones; with great restlessness and tossing about; the pulse, for the most part, less disturbed than could be supposed from the first sight of the patient; the tongue generally covered with a slimy yellowish coat; clamminess of the mouth, which in a few days, if the disease was not arrested, passed to a dry, brown, and in many cases verging to a black appearance.

There was mostly a remission of the fever, pain, &c. in the morning, but during the remission the patient would complain of extreme wretchedness, with considerable prostration of the vital powers. After this state continued three or four, and frequently seven or eight days, (but without any regularity in this respect,) an erysipelatous inflammation would become observable on some portion of the surface, often on the head and face, but frequently, on the extremities or some part of the body. When it came out fully, and remained on the surface, the case was generally more manageable than when it made its appearance in patches, for a short time, and then receded, which it was very apt to do. When this happened, the patients I believe rarely recovered. No matter what plan of treatment was pursued, the progress of the disease appeared to be onward; and the patient, after suffering almost indescribable torture, for perhaps ten or more days, sunk, worn out and exhausted by the efforts of nature to overcome her too powerful antagonist.

In these cases, when the efflorescence came to the surface and remained there, the patient generally got well, after a protracted course of treatment, varying from one week to three or four.

In the commencement of the epidemic, some practitioners made free use of the lancet; but this was afterwards discontinued from the fact, that some patients sunk rapidly after it; and in some others who ultimately recovered, the physician could not determine satisfactorily whether the bleeding was attended with any good; or whether it did not in reality do harm, by inducing prostration, which required all his energies and skill to overcome. All united in opinion, after becoming familiar with the disease, that venesection was a dangerous, or at least a very uncertain expedient.

The course of treatment found to be most efficacious was, emetics of ipecac. and tart. ant. combined; afterwards cathartics of calomel followed with jalap, or some other article in a few hours; and after free evacuations in this way, mild diluent drinks of whatever kind of herb teas was most convenient. Under this mild course of treatment, with the application of *raw cotton* to the inflamed surface, the patients were generally conducted to a safe and speedy cure.

Where, however, the physician was not called early, frequently it ran on from day to day until great prostration ensued, and sometimes the erysipelatous surface took on a dark, livid appearance, and gangrene, with extensive sloughing ensued.

Here, tonics, with powerful stimulants, became necessary. But this state of things was not apt to result, if medical aid was solicited in the commencement, and the above indicated anti-perturbating plan of treatment was at once instituted.

There are some cases of it lingering among us yet, though they are few. About three weeks ago I met with a very obstinate case of it, and I have a mild one on hand now. The former yielded to a steady perseverance in mild aperients and free diluents after emetics, repeated two or three times, and active purging in the commencement; and the latter is getting well under the same course of treatment, with the raw cotton to the inflamed leg.

Quite an unusual number of deaths occurred among us, but the most of them took place in the early part of the epidemic. This may be accounted for in two ways; in the commencement, people were not aware of the serious nature of the disease among us, and hence on becoming ill, deferred sending for aid until it had taken full possession of, and drawn into sympathetic disturbance, almost all the organs and functions of the system. It was a disease, too, the physicians had not met with before; and it was one well calculated to lead us into *too active* a course of treatment. Thus, all came to the conclusion that the violent pains attending severe cases, although preceded by severe chills, or rigors, were of a neuralgic character, and yielded to anodynes and sudorifics, better than to direct depletion by the lancet and antimonials, &c.

What tended, however, more than any thing else, perhaps, to do away the danger in most cases, was the state of the public mind. Every body became alarmed, and almost every one acquainted with a doctor, was inquiring "what they must do, if they or any of their family got it?" To these inquiries, my uniform reply was, "send for your physician as soon as you or they are attacked." And this course became generally pursued, and the fatal cases nearly disappeared.

